# Poverty, Child Labor, and Hope: The Relationship between Hope and Perceived Social Support in Children Working in Street Markets in TRC2 Region of Turkey

**DOI:** 10.3390/children7070078

**Published:** 2020-07-14

**Authors:** Mehmet Reşit Sevinç, Mustafa Hakkı Aydoğdu, Mehmet Cançelik, Turan Binici, Muhammed Ali Palabıçak

**Affiliations:** 1 Department of Bozova Vocational School, Harran University, 63850 Şanlıurfa, Turkey; 2 Department of Agricultural Economics, Faculty of Agriculture, Harran University, 63050 Şanlıurfa, Turkey; mhaydogdu@hotmail.com (M.H.A.); turanbinici@harran.edu.tr (T.B.); malipalabicak@harran.edu.tr (M.A.P.); 3 Department of Social Science Vocational School, Harran University, 63200 Şanlıurfa, Turkey; m.cancelik@harran.edu.tr

**Keywords:** poverty, child labor, social exclusion, hope, TRC2 Region, Turkey

## Abstract

Diyarbakır and Şanlıurfa (TRC2 Region) are the cities within the provinces of Turkey that have a high prevalence of poverty with an associated high child population. Due to the poverty in the cities of Diyarbakır and Şanlıurfa, this study investigated children working as a carrier in six purposefully selected districts, street markets, with the aim of providing social support for them to look forward to the future with hope. The research was conducted in 2019 by using questionnaires through face-to-face interviews, to cover all children in selected street markets within the scope of the full sampling volume. The data were analyzed using a structural equation modeling method. According to the results, 61.2% of working children are students, but most of them do not go to attend school; 8.7% of working children are the children of the Syrian refugees. Children work an average of 4.3 days a week and 8.6 h a day, with an average income of USD 1.6/day. As a result of the analysis, it was shown that the children received the most social support from their schoolmates (r = 0.428) to have hope in the future. This result shows that it is compulsory to include children in the education system. In this context, it is recommended that the financial contribution of children to their families should be paid by the state within the scope of social assistance and solidarity. Besides, strengthening social awareness and providing solidarity would contribute to the solution of the problem. The data obtained from this research could be used in studies and policies, to develop the concept of hope for combating poverty in regions with similar socio-economic characteristics. This research is the first of its type conducted on this issue in Turkey.

## 1. Introduction

Liberal and then neo-liberal economic policymakers in the history of the economy have argued that the free movement of labor and capital factors, the public sector shrinking, the private sector strengthening in economic activities, and the integration of national economies with the world economy (globalization) will benefit all countries. In particular, it has been argued that the self-regulation capacity of the free market economy will provide equal sharing of capital and other resources in the long run so that a fair distribution of wealth will be possible [1,2,3]. However, it is observed that the gap between rich and poor countries has been gradually growing after the 1990s. It is also noted that poverty increases, income distribution deteriorates and regional inequalities deepen with the economic policies implemented under the name of integration with the world economy, leading to foreign competition or structural adjustment policies. The limitation of neo-liberal policies and social state practices left the masses unprotected against economic changes, and unemployment became widespread after privatizations. While there is an increase in production and enrichment in a part of the world, on the other hand, poverty is increasing, and the events of hunger, lack of education, and violence that have emerged as an extension of these have kept the world agenda increasingly busy [1]. After the 1990s, the number of poor in the world population has increased day by day, and the social consequences of poverty have gradually deteriorated. One of the most important issues in the world after the 1990s is poverty. Globally, fighting poverty has become a common problem for all countries and communities [4]. In studies conducted, it is stated that poverty in the economy is seen as the underdevelopment problem, but poverty could not be eliminated in developed countries, as well [5,6]. On the other hand, even if a country does not have a poverty problem currently, it could be adversely affected by problems that are based on poverty problems in other countries.

In both academic and political debates, poverty emerges in two forms in the generally accepted basic definitions which are absolute poverty and relative poverty. Typically, relative poverty is seen as a matter of the failure of distributive justice, while absolute poverty is seen as a failure of meeting the requirements of the basic dignity of human beings or even a failure to meet human rights [7].

Absolute poverty refers to the situation in which individuals cannot reach the basic needs that must be met to sustain their lives. Such basic needs are often listed in international poverty reduction programs, and usually include food, water, shelter, basic education, and basic medical care [7,8]. In the definition of absolute poverty, it is expressed that a person could still lead a basic life. In other words, the available nutrition would provide the calories and other nutritional components that are necessary for him/her, biologically. Factors such as daily calorie requirement and how much of the income is spent on food are taken into account in establishing the absolute poverty line [9]. Those who have income that does not meet these basic needs are considered below the absolute poverty line [10,11,12]. Nevertheless, it has to be pointed out that absolute poverty, especially when it refers to high and middle-income countries, cannot be considered as a mere concept of serious resources deprivation or extreme poverty that puts individuals at risk of survival, as it is instead, for instance, with the USD 1.9 per day threshold set by the World Bank [13]. Rather, for developed countries, the absolute poverty threshold should be considered as a sort of “acceptable minimum” of living standards in the social context in which individuals live.

Relative poverty means poverty defined in comparison to other people’s standing in the economy. Thus a person can be poor in the relative sense, even if she/he is not poor in the absolute sense, that is, can meet her/his basic needs [7]. Relative poverty is a situation that emerges as a result of comparing one’s life level with a higher income group. Relative poverty can be observed by looking at relative standings within a society, or internationally [7,8]. The relative definition of poverty refers to the level of consumption and lifestyle required for a social person, not biologically. In this case, those who have income below the acceptable minimum consumption level in a particular society are defined as relatively poor [4,10,14,15]. The relative definitions that sociologists often adopt, on the other hand, show the resources of the individual or group compared to other members of the society, in other words, their relative living standards. Since relative poverty is a problem with inequalities in the distribution of resources in society, the poverty criteria could be said to be at least as objective as the absolute poverty criteria [16].

Since the 1970s, advanced capitalist democracies have entered a profound economic restructuring process. This restructuring process has also raised some social problems. Reconceptualization of basic definitions that determine social competence and satisfaction, such as social disadvantage, social support, vital hope, and poverty, also caused changes in moral perception. In some countries, Western welfare states [17,18], the emergence of poverty in terms of an emotional and sociological phenomenon rather than absolute economic one is expressed as the new poverty concept [18,19,20,21]. The 1980s were when the capitalist production process came to a restructuring stage, and when social problems surfaced. The literature agrees at this point, social inequalities could not be explained only by poverty, there is also a multidimensional process of deprivation and a new conceptualization should be made. Therefore, with this process, poverty moves away from an absolute and appears relatively [22,23,24,25,26].

Three paradigms nurtured relative poverty and were developed by Silver (1994). These are solidarity, specialization, and monopolistic paradigms [18]. The solidarity paradigm draws attention to the disappearance of the sense of “we” in the society, while the specialization paradigm draws attention to the “social class formation and differentiation” created by the economic division of labor. The monopolization paradigm draws attention to the fact that “democratic power is gathered in certain social groups and social inequalities emerge” [18,22,24].

In recent years, the world has made remarkable strides in advancing development. Yet, some 736 million people still live in extreme poverty. Children are disproportionately affected. Despite comprising one-third of the global population, they represent half of those struggling to survive on less than USD 1.90 a day. In almost every country in the world, richer and poorer countries, children are more likely to be living in poverty than adults, and everywhere, their particular life stage makes them more vulnerable to its devastating effects. Children who grew up impoverished often lack the food, sanitation, shelter, health care, and education they need to survive and thrive. Across the world, about one in every three children, roughly 663 million, live in households that are multidimensionally poor, meaning they lacked necessities as basic as nutrition or clean water [27,28].

The evaluation of child poverty as a separate area has emerged in parallel with the advocacy that children need special attention and protection, as they are vulnerable to poverty (and other environmental or societal hurdles) by virtue of their age and the level of their development. So, it is their age and level of their development that makes them vulnerable to poverty. Therefore, their rights should be regulated. It is also known that children’s perception of poverty is different from adults. These differences are an important factor in the separate assessment of child poverty [29,30,31]. It is inevitable for children in poverty to experience social exclusion. Social exclusion refers to a process that may differ depending on the period, regions, and socio-economic structure. The most fundamental component of this process is “not being able to participate in social life”; deprivation from one of the areas that ensure participation in social life constitutes a process that brings deprivation in other areas. For example, exclusion from the economic sphere could also lead to exclusion from social, political, or cultural processes [24,32]. 

There are two different discourses about child poverty which are well-becoming and well-being. While well-becoming focuses on the conditions and opportunities in which children will have a good future, well-being focuses on children having a happy childhood [33]. This research is focused on well-becoming. In this sense, it is important to be able to look at the future with hope for children who are surrounded by poverty and social exclusion. The typical dictionary definition of hope emphasizes the perception that something desired may happen [34]. Hope is the process of thinking about the goals of the individual together with “motivation to act, the agency” and “ways to achieve goals, the pathways” [35,36]. This definition formed the core of what has come to be called hope theory [35,37]. Hope is “expecting the best and working to achieve it” and “approaching life with excitement and energy” [38]. There are external and internal factors that increase hope in children. These are also the factors providing social support to the individual. 

Social support refers to the respect, caring, and help given by an important individual or another group that is close to an individual. Social support is not an obligation but a responsibility. Social support has been conceptualized as information leading the individual to believe that he or she is cared for and loved, esteemed and valued, and belongs to a network of communication and mutual obligation [39,40]. Social support could also be defined as the assistance based on responsibility, provided to the individual by the people around her/him. Individuals need to endure family members and friends who are seen as natural supporters in various periods. The support network created by these informal supports has a significant impact on the individual’s adaptation process and mental health [41]. 

Academic studies on social support were carried out primarily on the elderly and adults [42,43,44,45,46]. However, in recent years, academic studies on social exclusion experienced in children and adolescents, and their causes have started to increase [19,20,39,47,48,49]. Studies have found that there is a positive relationship between social support and welfare in adolescents and children [50,51]. Low education levels could be detrimental in a socio-economic sense and bring social exclusion of the individual [52,53]. A study has been conducted on the social incompatibilities of children of poor families in the USA. In the study, it was determined that children received social support from their older siblings and parents in overcoming the stress caused by social exclusion [20]. In a study on social support, change in children migrating from rural to the urban area in China concluded that the migrant students faced challenges and particular pressures, compared with their non-migrant peers, in terms of fulfilling their daily needs, because both their living arrangements and the available social support changed dramatically after migration [47]. In a study on children living on the streets in Nepal, it was found that children were away from their families due to poverty and received the social support they needed from their friends on the street. In the research, it was shown that children, despite everything dreamed about the future and believed that they could change their lives [54]. In a study conducted in the USA, it was shown that the students of African origin who are a minority have received social support mostly from the environment in which they are educated [50].

The average annual household of an individual disposable income equivalent in Turkey was 24,199 Turkish Lira (TL) in 2018. The average currency of USD 1 is equal to 4.82 TL in 2018 in Turkey [55]. The average annual equivalent household disposable income of the TRC2 region (Diyarbakır and Şanlıurfa) was 11,357 TL. There are 26 sub-regions in Turkey. The TRC2 region of Turkey has the third lowest ranking in terms of average annual equivalent household disposable income [56]. Turkey is composed of 81 provinces, the population was around 83.16 million at the end of 2019 [57]. According to the literature, the 0–17 age group is defined as a child population [58]. The child population accounted for 48.5% of the total population in Turkey in 1970, this ratio was 41.8% in 1990 and 27.51% in 2019. Şanlıurfa province has the highest ratio of the child population in 2019 in Turkey with 45.8%. The rate of the child population in Diyarbakır was 39.6% in 2019 [57,59]. The high share of Turkey’s overall child population in the provinces of Şanlıurfa and Diyarbakır (TRC2 Region) is due to the high fertility rate in these provinces. The total fertility rate refers to the average number of children a woman could have in the 15 to 49 age group. While the total average fertility rate was 2.07 children in 2017 in Turkey, this figure was 4.29 in Şanlıurfa and 3.31 in Diyarbakır in the same period. Şanlıurfa has the highest total fertility rate in Turkey [60].

According to the results of 2019 of the child labor survey of Turkey Statistical Institute (TUIK), the number of working children in the 5–17 age group in Turkey was 720,000 (4.4% of all children). While the employment rate of children in the 5–17 age group working in economic activity among children in the same age group was 4.4% of which 30.8% of them were working in agriculture, 23.7% of them in industry, and 45.5% of them in the service sector. The common reasons for children working were “helping the economic activity and income of the household” (59.1%), followed by “learning jobs, having a profession” (34.4%). Overall, 6.4% of the children work to “meet their own needs”, 66.1% of working children work in regular workplaces, 30.4% in fields and gardens, 3% in mobile unstable workplaces or street markets, and 0.5% at home [57,61]. Child workers working in street markets work independently on their account. During the shopping of the people coming to the market, they travel around with a wheelbarrow and take the products that these people bought back to their vehicles or their homes. In return, they charge a fee. This fee may differ depending on the time the buyer travels in the market, the weight of the products transported, the distance of transportation, and the transportation to the vehicle, or home, that is the place of delivery. 

There is still a gap in academic studies on social exclusion caused by child poverty and deprivation and the social support items children need to overcome it, and which of these elements positively affect the child’s hopes and expectations for the future. The scope of this study has been prepared to address this gap. In this study, children working as carriers in the districts where the normal and high-income groups live in Şanlıurfa and Diyarbakır were selected. Because in the districts with lower-income groups, customers carry the products they buy, there is almost no child labor in these street markets. The research started with the question of which districts do the individuals with good incomes of these cities live in? Then, it aimed to determine the relationship between the hopes and perceived social support of children working in the street markets of purposefully selected six districts and to reveal the individuals or groups that are effective in this context. Therefore, this study is the first study conducted in this regard in Turkey.

## 2. Materials and Methods

**Figure 1:**
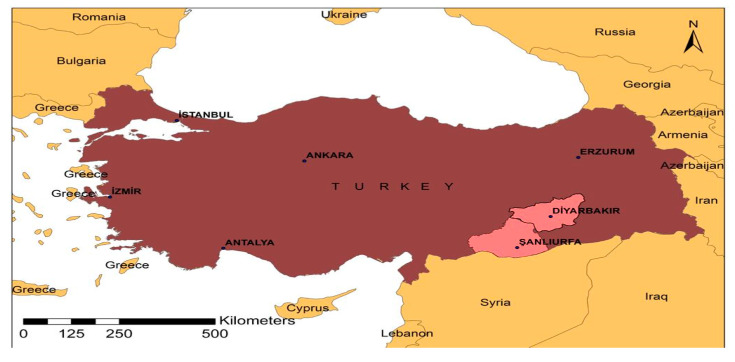
The location of Şanlıurfa and Diyarbakır in Turkey (Source: Prof. Dr. Mehmet Ali Çullu).

The research area is purposefully selected as the urban centers of Diyarbakır and Şanlıurfa provinces which are called as TRC2 region according to the TUIK in Turkey [62]. The economic poverty, total fertility rate, and high child population rate experienced by Şanlıurfa and Diyarbakır provinces were influential in the selection of the research area. Besides, these cities also have geopolitical significance due to take place in the Middle Eastern border of Turkey. The location of Şanlıurfa and Diyarbakır in Turkey is shown in Figure 1.

The main material of the study is the primary data from child workers who worked as carriers in street markets. Primary data is the type of data that is formed by collecting the researcher’s needs by using various tools in both qualitative and quantitative ways. Such data could be collected by three methods which are surveys, observations, and interviews [63]. The survey method was used in this research. Ethics committee approval and permission number 76244175-752.01.01 were obtained from Harran University Social and Humanities Ethics Committee on 13 April 2018 to conduct the prepared surveys and the research. The survey was conducted voluntarily with the consent of parents and children. The surveys were conducted in the street markets, where children are working by face to face interviews using the questionnaire when they are waiting for their customers. Before and after the surveys, observations were made by chatting with the children. The survey consists of three parts. The first part is the demographic characteristics, the second part is the hope scale, and the third part is the social support assessment scale. In this research, the dependent variable is hope and the independent variable is social support. Because the children surveyed are both working and studying, their hope-based social support resources are family members, schoolmates, and coworkers-colleagues. The hope scale was adapted in the survey questions from Snyder et al. (1997) [35], the social support scale was prepared by using Dubow and Ullman (1989) [64], and questions are given in the Appendix A (Table A1). The research was conducted in six districts in the urban centers of Diyarbakır and Şanlıurfa provinces on different days in 2019.

The sampling volume was not calculated, and with participation being voluntary, it was planned to survey with all children in the street markets during the survey with the full count sampling method. Twenty-seven of the children in the street market did not agree to conduct a survey. A total of 276 surveys were conducted: 126 children in Diyarbakır and 150 children in Şanlıurfa, who accepted the interview. It has been determined that some of the same children also work in street markets established on different days and in different districts. Therefore, special attention was paid not to have more than one interview with the same child in the research.

Structural equation modeling (SEM) was used to analyze the data collected through the survey. SEM is a general name given to techniques that allow examining hidden structures through observed variables [65]. A developed form of multiple regression model, which performs multiple regression analysis together, helps to define the relationships between observed and latent variables and enables hypotheses to be tested [66]. In a wider expression, SEM grows out of and serves purposes similar to multiple regression, but in a more powerful way which takes into account the modeling of interactions, non-linearities, correlated independents, measurement error, correlated error terms, multiple latent independents each measured by multiple indicators, and one or more latent dependents also each with multiple indicators. SEM may be used as a more powerful alternative to multiple regression, path analysis, factor analysis, time series analysis, and analysis of covariance. That is, these procedures may be seen as special cases of SEM, or, to put in another way, SEM is an extension of the general linear model of which multiple regression is a part [67].

## 3. Results and Discussion

The descriptive statistics of the children interviewed are shown in Table 1. The ages of the children who accepted the interview varied between 7 and 17 years. The average age of children that worked as carriers was calculated as 10.9 years. Of the carrier children, 1.4% were girls. This was a very striking and unexpected situation caused by the patriarchal family structure and region-specific culture of Şanlıurfa and Diyarbakır, meaning girls could not work as a carrier in the street markets. Girls were never encountered in a study on children working in street markets in Şanlıurfa, in 2010 [68]. All of these girls are children of Syrian refugee families. Most Syrian refugees come to Turkey because of the civil war that started in Syria in 2011. According to the Ministry of Internal Affairs Migration administration official records, there are 3.54 million Syrian refugees in Turkey. The number of Syrian refugees in Şanlıurfa is 423,820. In other words, about 12% of the Syrian refugees in Turkey are in Şanlıurfa. This rate corresponds to 20.72% of the population of Şanlıurfa [69]. Fifteen of the 27 children workers who did not agree to make interviews were Syrian refugee girls.

No street market is established in the research area on Sundays. From the sample, 6.1% of working children surveyed were orphans and 93.7% of the mothers of these children did not work. The rate of working fathers with (insured) regular employment mostly with minimum wage was 37.3%, the rate of temporary, daily, seasonal (uninsured) employees was 48.4%, and the rate of unemployed fathers was 14.3%. The average number of weekly working days of children was determined to be 4.3. The average working hours were from 9 am and the daily average working hours were determined as 8.6 h. The children stated that they especially waited until the end of the street market’s expiry period and that the shopkeepers distributed the leftovers of the goods they sold to the children for free. Weekly average declared earnings were 58 TL. It has been stated that the two things that make working children very happy are the extra money, that is, the tip, and the clothing aid given by the people. The two things that working children fear the most are the bullying tribute and theft of their wheelbarrows. Although 61.2% of children were students, it was observed that these children, work from morning to evening in the street markets established in different parts of the cities during the week. Therefore, these children are less likely to continue regular studies. In other words, the student status of these children is seen only as an official record. Although these children are not studying regularly at school, they are classed as students due to the compulsory education law in Turkey. There are criminal sanctions for families whose school-aged children do not enroll in school. Besides, the health, food, and clothing aids distributed by the public institutions from social aid and solidarity funds have an impact on the priorities given to families whose children go to school. Even if parents do not send their children to school to obtain these benefits, they officially enroll in the school. 

The average household size in the children’s homes was 7.7 people. The average household size was 3.4 persons in 2019 in Turkey according to TUIK data. The average household size was 5.4 in the same year in Şanlıurfa. Şanlıurfa after Şırnak (6.1 persons) in Turkey had the second largest average household size nationally [70].

To determine the relationship between hope and social support, the mean and standard deviations of the responses to the expressions given to the children with the five-point Likert scale are shown in Table 2. According to the results obtained from Table 2, the average of the family group where the working children get the most social support is 4.2/5. This is followed by 3.7/5 with schoolmates and 3.4/5 with colleagues in the street markets. The average of the child’s hope scale is 3.7/5. In the expressions of hope scale, the expression with the least participation was that the individual believed that she/he could find a way to solve their problems, while the most participation was in the expressions that education is important for the change of the individual life. 

It is seen that the eigenvalue of the 27 variables is greater than 1 which is given in Table 3 and is gathered under four factors explaining 58.484% of the total variance. Besides, the Kaiser–Meyer–Olkin (KMO) measure of sampling adequacy is 0.905. Bartlett Test is also significant at the level of 0.000, *p* < 1%. According to these results, it is possible to say that the data are suitable for factor analysis. The exploratory factor analysis results are given in Table 3.

The Cronbach alpha coefficient was used to determine the internal consistency of the expressions based on factors, in other words, their reliability. The Cronbach alpha coefficient ranges from 0 to 1. A negative value is an indication that the scale does not measure similar properties. The low alpha value indicates that the test is not homogeneous, which measures several properties together. A value between 0.60 and 0.80 indicates that the analysis is reliable, and a value above 0.80 indicates high reliability [71,72]. The values of these coefficients are shown in Table 4. The general Cronbach alpha coefficient for the expressions given below was measured as 0.907. This value indicates that the expressions are related to each other and are also very reliable for use in the analysis.

Table 5 contains the results of the model fit goodness. How well the model created in the SEM represents the data is determined by the goodness-of-fit indices. There are some certain critical limit points in each goodness-of-fit index but these are not certain, and are acceptable. Although there are a large number of the goodness-of-fit indices, only 5–6 of them are used in practice [73,74].

In determining the model fit, the chi-square goodness-of-fit index is checked first. The chi-square test is a test of the compatibility between the model and the data. The chi-square is not significant and X^2^/df ≤ 3 (according to some researchers X^2^/df ≤ 5) indicates the compatibility of the model. Even if the chi-square is meaningful, X^2^/df ≤ 3 indicates that the fit of the model is acceptable [75]. According to Table 4, chi-square value is high and statistically significant (X^2^ = 456.396, *p* = 0.000). However, the desired value is meaningless and has a lesser value. However, since the X^2^/df value is less than 3 (1435), it is possible to say that the model fits well for this index. Considering other fit indexes, the Incremental Fit Index (IFI) value is 0.959, the Comparative Fit Index (CFI) value is 0.958, the Normed Fit Index (NFI) value is 0.875, the Relative Fit Index (RFI) value is 0.852, the Parsimony Normed Fit Index (PNFI) value is 0.736, and the Parsimony Comparative Fit Index (PCFI) value is 0.806. The root mean square error of approximation (RMSEA) value is 0.040. According to these results, it could be said that the data provides sufficient representation [76,77,78].

**Figure 2:**
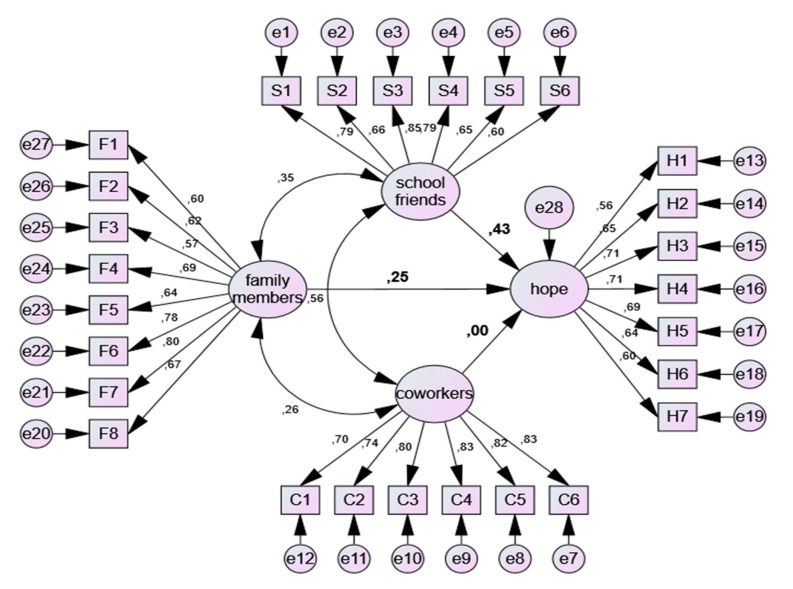
Structural Equation Model output.

As shown in Figure 2, the structural equation model output consists of four latent variables. Among these, the variables, family members, school friends (schoolmates), and coworkers (colleagues) are in the position of the extrinsic latent variable, while the hope variable is in the intrinsic latent variable. In Figure 2, it is shown that the external variables are connected to the internal variable by direction arrows as predictors. These arrows show the standardized regression coefficients.

The variables connected to the latent variables by the direction arrows are the observed variables. These are the measurements obtained during the data collection process. The relationship between the observed and latent variables is calculated by confirmatory factor analysis. As a result of the analysis, standard factor loads are calculated, showing the ability of the latent variable to calculate the observed variable. In this research, as shown in Figure 2, standard factor loads vary between 0.56 and 0.85. All of these values are statistically significant at *p* < 0.001 significance level.

Table 6 shows the standard regression coefficients and their significant values. According to the results, the effect of the support received from colleagues on the hope of the children could not be determined (*p* = 0.951 < 0.05). On the other hand, support from family members and schoolmates (*p* = 0.000 < 0.05) were found to have an impact on children’s hope. When the regression coefficients were evaluated, it was found that the support received from schoolmates (r = 0.429) had a stronger effect on the hope of children than the support received from family members (r = 0.248).

The sum of the emotions and thoughts that an individual imposes on herself/himself as an object reveals the self. Self is a combination of all of the individual’s assessments and beliefs about herself/himself [79]. The important people in the life of the child (family members, friends, etc.) are the primary elements that affect the formation of the concept of self [80]. It has been shown that the children interviewed do not receive social support from the other working children in the environment where they work, to have hope for the future. Competition in the environment in which children work has an impact on the emergence of this situation. Children who work as carriers in the streets market are forced to work due to poverty. Children contribute to household income with the earnings they earn by working all day long. Therefore, making money is of great importance for these children. This situation triggers competition in children. In the course of the field research, it was observed that the children applied or were exposed to bullying to catch customers. The following forms of bullying were determined: physical violence with 18.4%, psychological violence (abuse and threat) with 10.7%, and tributes with 6.8%. According to Atav and Baran (2020), Due et al. (2005) and Kumpulainen (2008), peer bullying in children occurs in every vital area [81,82,83]. Anxiety about earning income in the environment in which children work is a situation that triggers bullying. This situation in children working in street markets creates a self that constantly struggles with its peers, develops a defense mechanism against oppression, and uses its own bullying method whenever possible. 

A meaningful relationship was found between the social support received from the family members and the hope. However, the coefficient of this relationship (r = 0.248) is weak. The family environment is one in which the children live in a larger household with an average of 7.7 people. Türkdoğan (2008), Sevinç et al. (2018), and Davran et al. (2020) made some findings in their research on the community and family structure in this research area, such as, the minimal relationships of individuals, who are family members in the provinces of Diyarbakır and Şanlıurfa. Behaviors and words that show affection by family members are rarely used. Household authority is concerned with trying to provide the family with continuous protection against the external environment. Poverty in the household strengthens this protection instinct because the head of the family only has his family members as support and so he must protect them [5,84,85]. Therefore, the social support that children receive from their family members to look forward to the future is weak.

A significant relationship has been shown between the social support that children receive from their schoolmates and hope, and the strength of this relationship (r = 0.429) is higher than the support received from the family. Dubow and Ullman (1989), Elias and Haynes (2008), Chu et al. (2010), Wu et al. (2017) found that children receive strong social support from school settings in their studies [19,47,50,64]. Children beginning at age 6 may have 12 years of compulsory education system in Turkey [86]. Although children work during school periods, they do not want to break their ties with the school. Social support that they receive from their schoolmates enables them to have hope for the future.

## 4. Conclusions

The research area, Diyarbakır, and Şanlıurfa (TRC2 Region) are the provinces in which poverty is the most severe in Turkey. In these provinces, the proportion of the child population in the total population is also quite high. This situation forces children to work when they are old enough to work, to contribute to the household income. The monthly disposable income of those living in the TRC2 region was USD 196.35 in 2019. The contribution of these children to the monthly average family budget is 232 TL (USD 48.13), in other words, USD 1.6 per day. This amount of money is important for families.

Social support is very important for the physical and spiritual development of children and enables them to hold on to life with more hope. The social support needed is received primarily from the family members who represent a child’s internal environment and then from the external environment outside the family. Although children get the most social support from the family, the school environment and schoolmates come to the fore in the relationship of hope and social support in the research area. Children are aware that they can get rid of the clamp of poverty through education. In other words, education is an important source of support that will create hope for the future for them. They are also aware of the positive change that the education they receive in the school environment would create in themselves. They stated that their friends and teachers at the school value and listen to them and help them to solve their problems. While the least participation on the hope scale was the belief that the child could find a way to solve their problems, the most participation was in the expressions that education is important for the change of her/his life. All these factors cause children to receive more hopeful social support from the school environment and schoolmates rather than from their family members and friends in the environment in which they work.

Children should not move away from the school environment and should continue their education to have hope for the future. There is a 12-year compulsory education system that begins from the age of 6 in Turkey. However, the children in the research area work 4.3 days a week and 8.6 h a day, carrying loads with hand carts in the street markets. They could not attend school due to this heavy child labor. This shows that the existing legal regulations and inspections are insufficient. Therefore, it is necessary to revise the legal regulations and strengthen them by increasing the controls. However, this measure alone would not be sufficient because the reasons for not including children in the education system should be eliminated. In other words, poverty in households where these children live should be eliminated. This is possible with macroeconomic policies that increase regional development. However, the implementation of macro policies requires a high investment budget and it takes time to see the positive effects. 

The easiest and fastest solution is for the state to provide the financial contribution that children make to their households by working for USD 48.13 per month, within the scope of social assistance support for their families without the need for children to work by the state. This is possible with various transfer expenditures and subsidies made within the framework of social state understanding. One of the unexpected and encountered situations during the research was the Syrian refugee children working in street markets. Their ratio in the sample was 8.7%. In this context, international charities also have duties. Preventing or reducing heavy child labor at a young age is one of the global goals. 

On the other hand, strengthening social sensitivity and ensuring social solidarity is also important for children working as carriers in the street markets. This solidarity would cause all working children to have more hope in the future. However, this situation is not a necessity but a responsibility for both internal and external environments that provide social support to children. In developed societies, individuals are more aware of this responsibility, while in less developed societies this awareness needs to be increased. Therefore, more regional, national, and global solidarity and aid should be provided for these children. This research is the first study carried out in terms of revealing the relationship between hope and social support in Turkey. The results provide useful data to researchers, decision-makers, and policymakers working on these concepts. In addition, these results could be used in regions and countries with similar socio-economic characteristics.

## Figures and Tables

**Table 1:** The descriptive statistics of the participants.

Variable	Definition	Mean	Std. Dev
Age	Year	13.37	1.941
Gender	If female 0, (1.4%); male is 1 (98.6%)	0.99	0.120
Education	1 for illiterate (4%), 2 for primary school students (5.8%), 3 for primary school graduates (3.6%), 4 for secondary school students (55.4%), 5 for secondary school graduates (31.2%)	3.46	1.290
Household Number	The household number of the family	7.70	3.283
Number of Employees in the family	Number of individuals working in different jobs in the family	3.17	2.679
Type of residence	1 for non-family (with strangers) (2.9%), 2 for extended family (26.4%), 3 for nuclear family (70.7%)	2.68	0.527
Hometown	1 for Syrian refugee (8.7%), 2 for Şanlıurfa (47.1%), 3 for Diyarbakır (44.2%)	2.35	0.635
Rural migration	If yes 1 (63.0%), no is 0 (37.0%)	1.36	0.483
Connection with the rural	If yes 1 (67.8%), no is 0 (32.2%)	1.32	0.468

**Table 2:** Averages and standard deviations for the expressions.

Expressions	Mean	Std. Dev.	Expressions	Mean	Std. Dev.
H1	3.60	1106	S1	3.78	1.248
H2	3.63	1277	S2	4.08	1.155
H3	3.52	1133	S3	3.79	1.067
H4	3.62	1137	S4	3.37	1.332
H5	3.37	1329	S5	3.16	1.256
H6	3.80	1190	S6	3.89	1.161
H7	4.00	1289	F1	4.29	1.059
C1	3.79	1096	F2	4.40	0.870
C2	3.19	1239	F3	3.63	1.222
C3	3.34	1248	F4	4.17	0.861
C4	3.53	1155	F5	4.47	0.978
C5	3.14	1338	F6	4.11	0.966
			F7	4.32	0.883
			F8	4.24	0.999

H: hope, C: colleagues-social support; S: schoolmates-social support; F: family members-social support.

**Table 3:** The exploratory factor analysis.

Component							
Family Members (8.083) *		Coworkers-Colleagues (3.422) *		Hope (2.597) *		Schoolmates (1.689) *	
F7	0.790	C6	0.836	H4	0.757	S3	0.792
F6	0.784	C4	0.831	H5	0.752	S1	0.764
F1	0.725	C3	0.821	H3	0.730	S4	0.724
F4	0.697	C5	0.783	H6	0.713	S5	0.700
F5	0.679	C1	0.759	H2	0.629	S2	0.677
F2	0.676	C2	0.755	H7	0.603	S6	0.643
F8	0.668			H1	0.566		
F3	0.648						

* Initial Eigenvalue.

**Table 4:** Reliability analysis of expressions based on factors.

Factors	Cronbach Alfa
Hope	0.837
Support from colleagues	0.906
Support from schoolmates	0.866
Support from family members	0.864

General Cronbach Alfa: 0.907.

**Table 5:** The goodness-of-fit results of the model.

Fit Indices	Model
X^2^ (Chi-Square) value	456.396
Degree of freedom (df)	318
*p* (Level of Significance)	0.000
X^2^/df	1.435
Incremental fit index, IFI	0.959
Comparative fit index, CFI	0.958
Normed fit index, NFI	0.875
Relative fit index, RFI	0.852
Parsimony Normed Fit Index, PNFI	0.736
Parsimony Comparative Fit Index, PCFI	0.806
Root Mean Square Error of Approximation, RMSEA	0.040

**Table 6:** Standard regression coefficients.

Regression Direction	Regression Coefficient	Level of Significance
Hope ← Coworkers-Colleagues	0.005	0.951
Hope ← Family members	0.248	0.000 *
Hope ← School friends-Schoolmates	0.429	0.000 *

* It shows the regression coefficients at the 0.001 significance level.

## Appendix A

**Table A1:** The hope and social support scale questions used in the survey

Hope Scale	
H1	I can think of many ways to get things that are very important to me in life.
H2	I am as good as children of the same age as me.
H3	When I have a problem, I can find many ways to solve this problem.
H4	I think what I did in the past will help me in the future.
H5	I know that I can find methods or ways to solve the problem, even if others want to give up.
H6	I believe that my life will change in a good direction in the future.
H7	I think education is important for my life to change
**Social Support Scales**	
**Social support from coworkers-colleagues**	
C1	Are you loved by your friends?
C2	Do your friends like to listen to your thoughts?
C3	Do you and your friends do a lot for each other?
C4	Do you feel very close to your friends?
C5	Do you trust your friends to get help or advice when you have problems?
C6	Do you think your friends care about you?
**Social support from schoolmates**	
S1	Do you feel like part of the class?
S2	Do you feel that you are excluded by the class?
S3	Are you loved by your classmates?
S4	In the classroom, do children do a lot for each other?
S5	Do your classmates help you when you have problems?
S6	Will your classmates make you feel bad?
**Social support from family members**	
F1	Can you trust your family to get help or advice when you have problems?
F2	Do you and your family do a lot for each other?
F3	Would you share a lot with your family?
F4	When you need them, do you feel that your family is with you?
F5	Do you feel that you are excluded by your family?
F6	Do you have an important place in your own family?
F7	Do you think your family cares about you?
F8	Do you feel like part of the family?
